# Fibrinolytic system activation immediately following trauma was quickly and intensely suppressed in a rat model of severe blunt trauma

**DOI:** 10.1038/s41598-021-99426-2

**Published:** 2021-10-13

**Authors:** Mineji Hayakawa, Takumi Tsuchida, Yoshinori Honma, Asumi Mizugaki, Takayoshi Ooyasu, Tomonao Yoshida, Tomoyo Saito, Kenichi Katabami, Takeshi Wada, Kunihiko Maekawa

**Affiliations:** Department of Emergency Medicine, Hokkaido University Hospital, N14W5, Kita-ku, Sapporo, 060-8648 Japan

**Keywords:** Haematological diseases, Trauma

## Abstract

In severe trauma, excessive fibrinolytic activation is associated with an increase in the transfusion volume and mortality rate. However, in the first several hours after a blunt trauma, changes in fibrinolytic activation, suppression, and activation–suppression balance have not yet been elucidated, which the present study aimed to clarify. Anesthetized 9-week-old male Wistar S/T rats experienced severe blunt trauma while being placed inside the Noble–Collip drum. Rats were randomly divided into four groups of seven. The no-trauma group was not exposed to any trauma; the remaining groups were analysed 0, 60, and 180 min after trauma. Immediately following trauma, total tissue-plasminogen activator (tPA) levels significantly increased in the plasma, and the balance of active tPA and active plasminogen activator inhibitor-1 (PAI-1) significantly tipped toward fibrinolytic activation. After trauma, both tPA and PAI-1 levels increased gradually in various organs and active and total PAI-1 levels increased exponentially in the plasma. Total plasma tPA levels 60 min after trauma returned quickly to levels comparable to those in the no-trauma group. In conclusion, fibrinolytic activation was observed only immediately following trauma. Therefore, immediately after trauma, the fibrinolytic system was activated; however, its activation was quickly and intensely suppressed.

## Introduction

In severe trauma, excessive fibrinolytic activation is associated with an increase in the transfusion volume and mortality rate^1–5^. Many investigations have reported that excessive fibrinolytic activation is observed in patients immediately following severe trauma^3–5^. Early administration of tranexamic acid, an anti-fibrinolytic agent, to severe trauma patients has improved the mortality rates in large international randomized control trials^6–8^, in which tranexamic acid was administered continuously during the first 8 h after trauma. However, changes in the fibrinolytic system during the first several hours after severe trauma have not been sufficiently elucidated.

Tissue-plasminogen activator (tPA) may play a central role in excessive fibrinolytic activation immediately following trauma in patients with severe trauma^1,9^. In the fibrinolytic system, tPA triggers fibrinolytic activation to convert the proenzyme plasminogen into the proteinase plasmin^10^. Large amounts of tPA are stored in granules of vascular endothelial cells throughout the body and are released acutely for a rapid increase in blood tPA levels in response to various stimulations, including coagulation activation^10^. The released tPA then activates plasminogen to plasmin, which degrades fibrinogen and fibrin^10^. This fibrinolytic system is regulated by three distinct inhibitors comprising plasminogen activator inhibitor-1 (PAI-1), α_2_ plasmin inhibitor (α_2_PI), and thrombin-activatable fibrinolysis inhibitor (TAFI)^11–13^.

At the initial fibrinolytic step to activate plasminogen to plasmin via tPA, PAI-1 regulates free active tPA^11^. PAI-1 is mainly synthesized in vascular endothelial cells and hepatocytes by various stimulations (interleukin-1^14^, tumour necrosis factor-α^15^ and thrombin^16^, etc.) and is secreted into the plasma^17^. Active PAI-1 can rapidly inhibit tPA activity by binding 1:1 with free active tPA, resulting in the formation of the tPA-PAI-1 complex^11^. After forming the tPA-PAI-1 complex, the activities of both tPA and PAI-1 are irreversibly lost^11^. After plasmin generation, α_2_PI can rapidly inhibit plasmin by binding 1:1 with plasmin, resulting in the formation of the plasmin-α_2_PI complex (PIC)^12^. TAFI is converted to activated TAFI (TAFIa) by thrombin, and TAFIa reduces the binding of plasminogen on the fibrin surface by partial degradation of fibrin^13^. As a result, activation of plasminogen to plasmin by tPA on the fibrin surface strongly reduces^13^.

In the acute phase of severe blunt trauma, temporal changes in fibrinolytic activation, suppression, and the balance between activation and suppression have not yet been elucidated. Our hypothesis is that although the fibrinolytic system is activated immediately following trauma, the activation does not continue for a long duration and is supressed quickly. Therefore, the present study aimed to use a severe blunt trauma rat model to clarify the temporal changes in fibrinolytic activation, suppression, and the balance between the two during the first several hours after trauma.

## Methods

### Animals

Nine-week-old male Wistar S/T rats were obtained from Japan SLC (Hamamatsu, Japan). All animal procedures were approved by the Institutional Ethical Review Board of Hokkaido University. All rats were housed and treated in accordance with the standards of animal experiments at Hokkaido University. The study was carried out in compliance with the ARRIVE guidelines. Animals were allowed to acclimate for several days at our animal breeding quarters before being subjected to experimentation. The breeding quarters were maintained at 20 °C on a 12-h light/dark cycle. The animals were provided ad libitum access to a standard diet and water. One day before the experiments, the animals were housed in the fasted state but provided ad libitum access to water.

### Experimental procedures

Twenty-eight rats (body weight, 280–320 g) were anesthetized by a combination anesthetic prepared with 0.375 mg/kg of medetomidine, 2.0 mg/kg of midazolam, and 2.5 mg/kg of butorphanol^18^. During the experimental period, the rectal temperature was maintained at 37–39 °C. After the rats were anesthetized, they were restrained in the supine position. A tracheostomy was performed using a small incision, and the left carotid artery and right external jugular vein were exposed. The rats were randomly divided into four groups of seven rats each: no-trauma group, 0 min group, 60 min group, and 180 min group.

**Figure 1:**
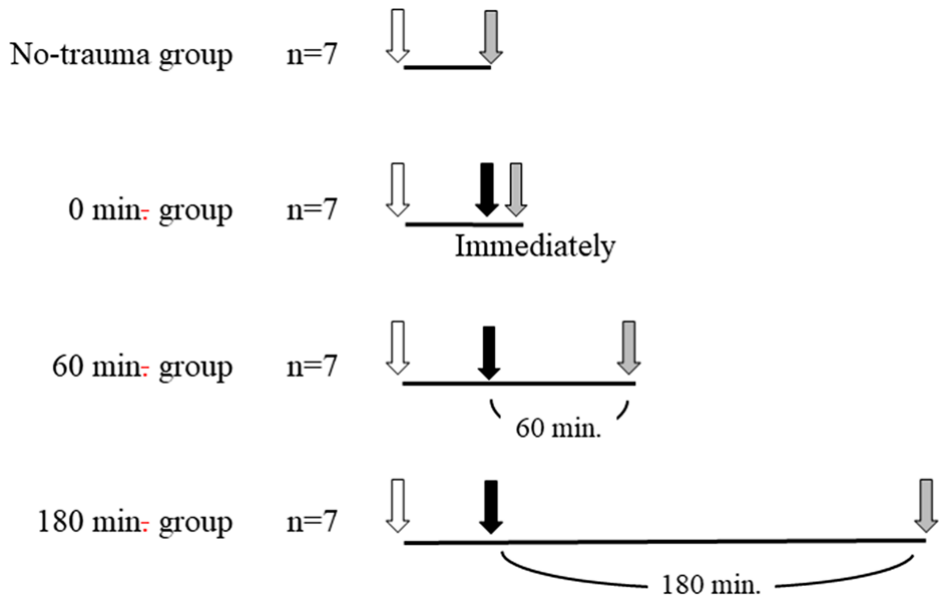
The schema of the experimental procedures. In no-trauma group, blood and tissue samples were collected following anesthesia. In the 0 min group, 60 min group and 180 min group, following anesthesia, severe blunt trauma was inducted. In the 0 min group, blood and tissue samples were collected immediately following induction of trauma. In the 60 min and 180 min group, blood and tissue samples were collected 60 and 180 min following induction of trauma, respectively. White arrow, anesthesia; black arrow, induction of severe blunt trauma; grey arrow, mean arterial pressure recorded and samples collection.

In the no-trauma group, mechanical ventilation using SERVO 900C (FUKUDA DENSHI, Tokyo, Japan) was initiated via tracheostomy. Mechanical ventilation was set as the pressure control mode with FiO_2_ = 0.4, PEEP = 4 cmH_2_O, pressure control = 15 cmH_2_O, and respiratory rate = 80/min. The left carotid artery was immediately catheterized with a 24-gauge SURFLO catheter (Terumo, Tokyo, Japan) to permit mean arterial pressure monitoring and arterial blood sampling. The mean arterial pressure was monitored using a TruWave Disposable Pressure Transducer (Edwards Lifesciences, Irvine, CA, USA) and a Viridia component monitoring system (Hewlett–Packard Japan, Tokyo, Japan). To maintain arterial catheter patency, normal saline (19 mL) with 3.2% sodium citrate solution (1 mL) was constantly infused at 2 mL/h. Furthermore, the right external jugular vein was immediately catheterized using a silicone microtube. After these procedures, the mean arterial pressure was recorded. Furthermore, blood sample and tissue samples from the lung, liver, and kidney were collected (Fig. 1).

In the 0 min group, 60 min group, and 180 min group, rats were subsequently placed in the Noble–Collip drum, a plastic wheel 38 cm in diameter with internal shelves (supplementary figure) and rotated for 500 revolutions at 50 rpm^19–21^. During rotation, the anesthetized rat was repeatedly struck down from the top of the drum’s interior^19–26^. Even though each hit is not completely same, the total severity of trauma is averaged in each rat since the model receives many hits. This trauma model is a quantitative, severe blunt trauma model without massive bleeding^19–26^. In the present study, we confirmed no massive intraabdominal and intrathoracic bleeding on sampling of the organs. After induction of severe trauma, mechanical ventilation, catheterization into the left carotid artery and right external jugular vein were performed similar to the no-trauma group. In the 0 min group, blood and various tissue samples were collected immediately. In the 60 min and 180 min group, observation durations were 60 and 180 min following the induction of severe trauma, respectively. During the observations, Ringer’s lactate solution was continuously administered to maintain a mean arterial pressure of 60 mmHg via the right external jugular vein. Following the observations, the mean arterial pressure was recorded and, blood and various tissue samples were collected (Fig. 1).

### Blood and tissue sample collections

Blood samples were immediately diluted with 3.2% sodium citrate (1:9 v/v). A portion of the whole blood sample was used for arterial blood gas analysis. The remainder of the blood sample was separated through serial centrifugation (15 min at 3,500 rpm at 25 °C, twice). The supernatant was collected and frozen at − 80 °C until analysis. Tissue samples from the lung, liver, and kidney were immediately collected and immersed in RNAlater (QIAGEN K. K., Tokyo, Japan). The tissue samples in RNAlater were incubated at 4 °C overnight and stored at − 80 °C after removal from the RNAlater.

### Real-time quantitative polymerase chain reaction (RT-PCR)

To detect the mRNA expression of PAI-1 and t-PA in the lung, liver, and kidney after various observation periods, we performed real-time RT-PCR. Total RNA was extracted from tissue samples using the RNeasy Lipid Tissue Mini Kit (QIAGEN K. K.) according to the manufacturer’s instructions. To eliminate contaminating DNA, an RNase-Free DNase Set (QIAGEN K. K.) was used. RNA (1 μg) was reverse transcribed using the SuperScript® VILO™ cDNA Synthesis Kit (Thermo Fisher Scientific K.K, Tokyo, Japan) in a final volume of 20 μL. A volume of 1.25 μL of the reverse transcription reaction was amplified using the TaqMan® PreAmp Master Mix (Thermo Fisher Scientific K. K). Primers for rat SERPINE1 (PAI-1; Rn01481341_m1), rat PLAT (t-PA; Rn01482578_m1), and rat GAPDH (glyceraldehyde-3 phosphate dehydrogenase; Rn01775763_g1) as an endogenous control were purchased from TaqMan® Gene Expression Assay (Thermo Fisher Scientific K. K.). Real-time PCR was performed using TaqMan® Universal Master Mix II, no UNG (Thermo Fisher Scientific K. K.), and BioMark™ 96.96 Dynamic Array (Fluidigm K. K., Tokyo, Japan). The results of RT-PCR were analyzed using Fluidigm Real Time PCR Analysis 3.0.2 (Fluidigm K. K.) to calculate C_t_ values. GAPDH values were used to normalize the data. A relative quantitation method [ΔΔC_t_] was used to evaluate the expression of each gene relative to that of the control^27^. ΔC_t_ of the calibrator was defined as the mean ΔC_t_ in the control group. All procedures were performed according to the manufacturer’s instructions.

### Blood sample measurements

Arterial blood gas analysis was performed using ABL 700 (Radiometer, Tokyo, Japan). Soluble fibrin and α2PI were measured using the latex agglutination test and synthetic substrate assay, respectively, using the STACIA CN10 instrument (LSI Medience Corporation, Tokyo, Japan). Active PAI-1 antigen, total PAI-1 antigen, active tPA antigen, total tPA antigen, plasminogen antigen, and plasmin α_2_PI complex antigen were measured using the Rat PAI-1 Activity ELISA Kit, Rat PAI-1 Total Antigen ELISA Kit, rat tPA activity ELISA kit, rat tPA total antigen assay ELISA kit (all from Molecular Innovations, Inc., MI, USA), rat plasminogen ELISA kit (Abcam, Cambridge, UK), and rat plasmin-antiplasmin complex (PAP) ELISA kit (Wuhan Huamei Biotech Co., Ltd., Wuhan, China), respectively. The rat PAI-1 Total Antigen Assay ELISA Kit cannot discriminate free PAI-1 from the tPA·PAI-1 complex, whereas the rat tPA Total Antigen Assay ELISA kit cannot discriminate free tPA from the tPA·PAI-1 complex. All procedures were performed according to the manufacturers’ instructions. The description of the measurement components of the present study are presented in Table 1.

**Table 1:** Components of coagulation and fibrinolytic system measured in the present study.

Components	Description
Soluble fibrin	It is generated after cleaving fibrinogen by thrombin and its elevation indicates coagulation activation
**Plasminogen activator inhibitor 1 (PAI-1)**	It is a principal inhibitor of active tPA
Active PAI-1	It is PAI-1 which is able to inhibit active tPA by binding at 1:1
Total PAI-1	It includes active PAI-1 and in-active PAI-1 (latent form and tPA·PAI-1 complex)
**Tissue- plasminogen activator (tPA)**	It has a central role in fibrinolytic system by activating plasminogen to plasmin
Active tPA	It is tPA which is able to activate plasminogen to plasmin
Total tPA	It includes active tPA and tPA·PAI-1 complex
Plasminogen	It is a zymogen of plasmin, which is the major enzyme that degrades fibrin clots
α_2_-plasmin inhibitor (α_2_PI)	It is a primary and fast inhibitor of plasmin, which is an important enzyme to degradate fibrin clots
Plasmin α_2_PI complex (PIC)	It is a complex of plasmin and α_2_PI. Its elevation indicates production of plasmin

### Statistical analyses

Unless otherwise indicated, all measurements are expressed as the mean ± standard deviation. Comparisons among the four groups were performed using one-way ANOVA with the Dunnett’s multiple comparison test. Comparisons of mRNA expression of tPA and PAI-1 among the four groups were performed using two-way ANOVA. SPSS 25 (IBM Japan K. K., Tokyo, Japan) was used for all statistical analyses. The level of statistical significance was set at *P* < 0.05.

## Results

The general characteristics and coagulation activation of each group are shown in Table 2. Immediately following the severe blunt trauma, a decrease in arterial blood pressure, lactic acidosis, and haemoconcentration, which would result from vascular permeability induced by massive tissue damage, were observed. Over time, the lactic acidosis improved. Although infusion of Ringer’s lactate solution was needed to maintain the mean arterial pressure, haemodilution was not observed even 180 min after trauma induction. Soluble fibrin levels, indicating coagulation activation, gradually increased after trauma (*P* < 0.001, one-way ANOVA). The soluble fibrin level 180 min after trauma was higher than that in the no-trauma group (*P* < 0.001).

**Table 2:** General characteristics and coagulation activation of each group.

	No-trauma	Trauma			P value
		0 min	60 min	180 min	
	n = 7	n = 7	n = 7	n = 7	
Mean arterial blood pressure (mmHg)	137 ± 17	83 ± 19***	76 ± 7***	96 ± 19***	< 0.001
Hemoglobin (g/L)	14.9 ± 1.4	16.5 ± 1.1	16.5 ± 1.9	15.3 ± 1.3	0.108
Lactate (mmol/L)	0.54 ± 0.11	4.67 ± 1.21***	2.53 ± 1.08**	2.16 ± 1.42*	< 0.001
Infusion volume (mL/kg)	0 ± 0	0 ± 0	28 ± 38	39 ± 23**	0.004
Soluble fibrin (μg/mL)	25.3 ± 24.5	63.7 ± 11.6	101.3 ± 65.6	332.3 ± 102.7**	< 0.001

No-trauma and 0 min groups were not infused with Ringer’s lactate solution. P values are obtained using one-way ANOVA. ***P < 0.001 using Dunnett’s test for the no-trauma group; **P < 0.01 using Dunnett’s test for the no-trauma group; *P < 0.05 using Dunnett’s test for the no-trauma group.

### tPA and PAI-1 levels in the plasma

**Figure 2:**
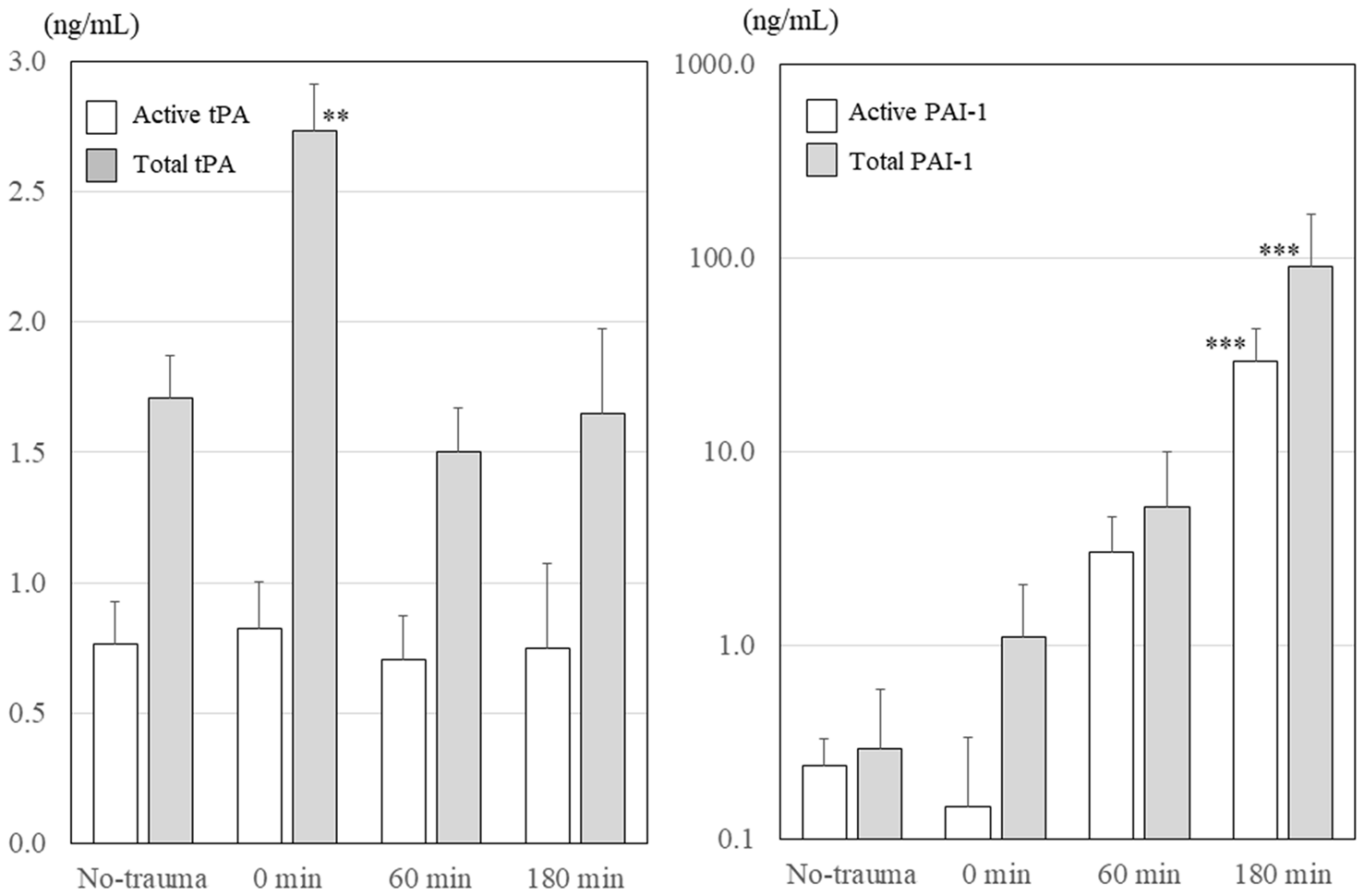
Changes in plasma tPA and PAI-1 levels. Although total tPA levels increased immediately after trauma (0-min group) (*P* < 0.001, one-way ANOVA), active tPA levels did not change after trauma. Although active PAI-1 levels decreased immediately after trauma (0-min group), active PAI-1 levels increased gradually (*P* < 0.001, one-way ANOVA). Active and total PAI-1 levels gradually increased to more than 100 times the levels of the no-trauma group 180 min after trauma (*P* < 0.001, one-way ANOVA). tPA, tissue-plasminogen activator; PAI-1, plasminogen activator inhibitor-1. ***P < 0.001 using Dunnett’s test for the no-trauma group; **P < 0.01 using Dunnett’s test for the no-trauma group.

Changes in the active and total tPA levels in the plasma are presented on the left panel of Fig. 2. The total tPA level immediately following trauma (0-min group) significantly increased (*P* < 0.001, one-way ANOVA; *P* < 0.01, post-hoc Dunnett’s test). However, active tPA levels did not change. Changes in the total and active PAI-1 levels in the plasma are presented in the right panel of Fig. 2. Although active PAI-1 levels decreased immediately following trauma, the active and total PAI-1 levels in the 180-min group were several hundred times higher than levels in the no-trauma group (*P* < 0.001, one-way ANOVA).

### Balance between activation and suppression of the fibrinolytic system

**Figure 3:**
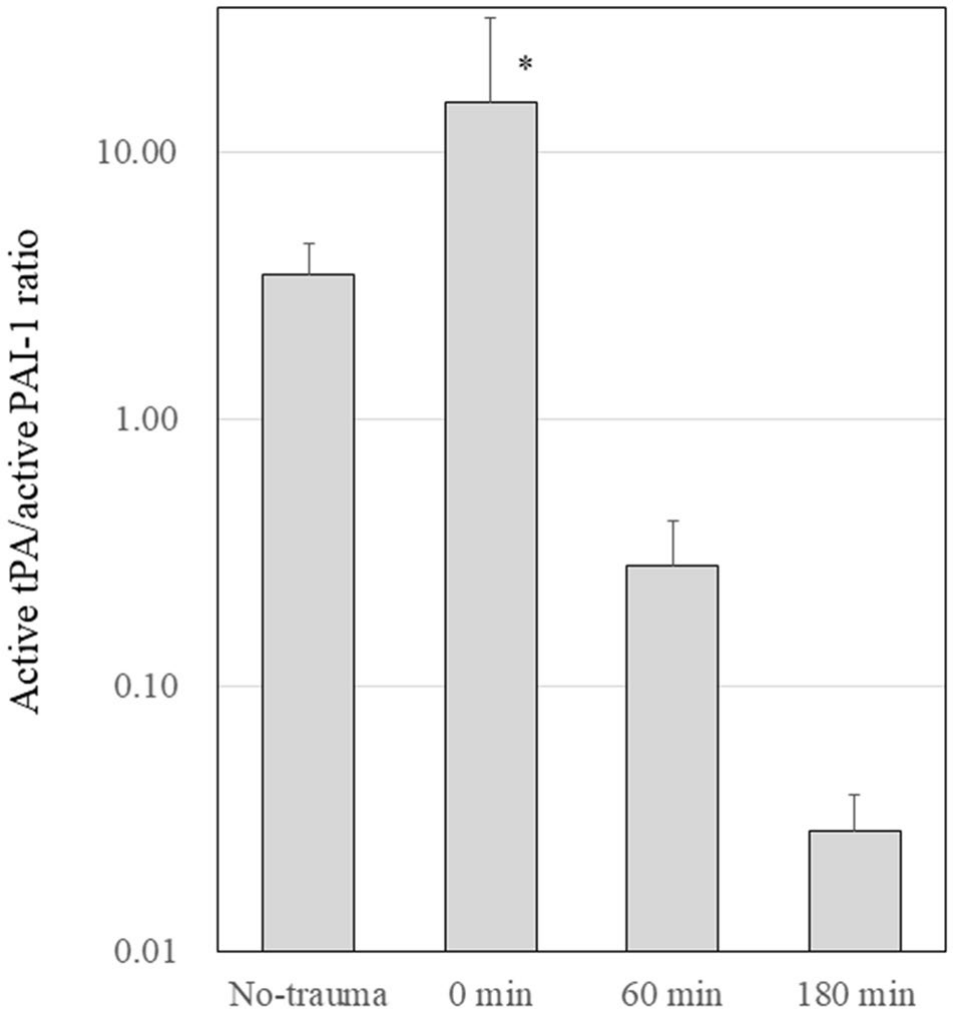
Balance between activation and suppression of the fibrinolytic system. Active tPA and active PAI-1 ratios are presented. Although the balance tipped toward fibrinolytic activation immediately after trauma, the balance tipped toward fibrinolytic suppression at 60 and 180 min (*P* < 0.001, one-way ANOVA). tPA, tissue-plasminogen activator; PAI-1, plasminogen activator inhibitor-1. *P < 0.05 using Dunnett’s test for the no-trauma group.

Figure 3 presents the balance between active tPA and active PAI-1 in the plasma. Although the balance tipped toward fibrinolytic activation immediately following trauma, the balance tipped toward fibrinolytic suppression at 60 and 180 min (*P* < 0.001, one-way ANOVA).

### mRNA expression of tPA and PAI-1

**Figure 4:**
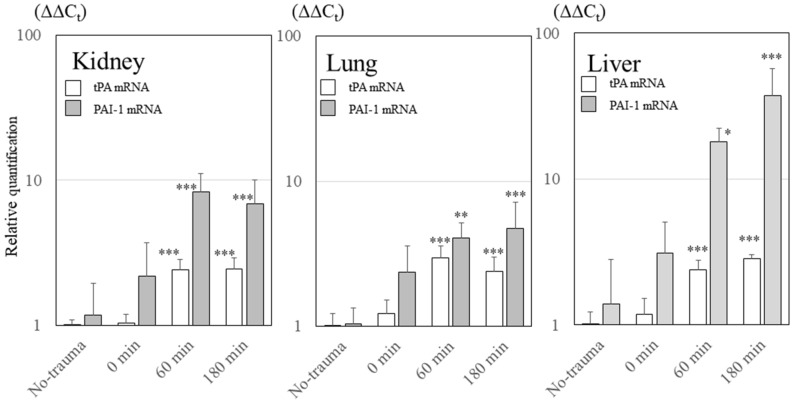
Changes in tPA and PAI-1 mRNA expression levels in various organs. The mRNA expression levels of both tPA and PAI-1 gradually increased after trauma in all organs (all comparisons revealed *P* <  0.001, one-way ANOVA). The increase in PAI-1 mRNA expression was higher than that of tPA mRNA (in kidney, *P* <  0.001; in liver, *P* < 0.001; in lung, *P* = 0062; two-way ANOVA). tPA, tissue-plasminogen activator; PAI-1, plasminogen activator inhibitor-1. ***P < 0.001 using Dunnett’s test for the no-trauma group; **P < 0.01 using Dunnett’s test for the no-trauma group; *P < 0.05 using Dunnett’s test for the no-trauma group.

Figure 4 shows changes in the mRNA expression levels of tPA and PAI-1 in the kidney, lung, and liver. The mRNA expression levels of both tPA and PAI-1 gradually increased in all organs after trauma (all comparisons revealed *P* < 0.001 by one-way ANOVA). The increase in PAI-1 mRNA expression was higher than that of tPA mRNA (in kidney, *P* < 0.001; in lung, *P* = 0.062; in liver, *P* < 0.001; two-way ANOVA).

### Plasminogen, α_2_ plasmin inhibitor, and PIC levels in the plasma

**Figure 5:**
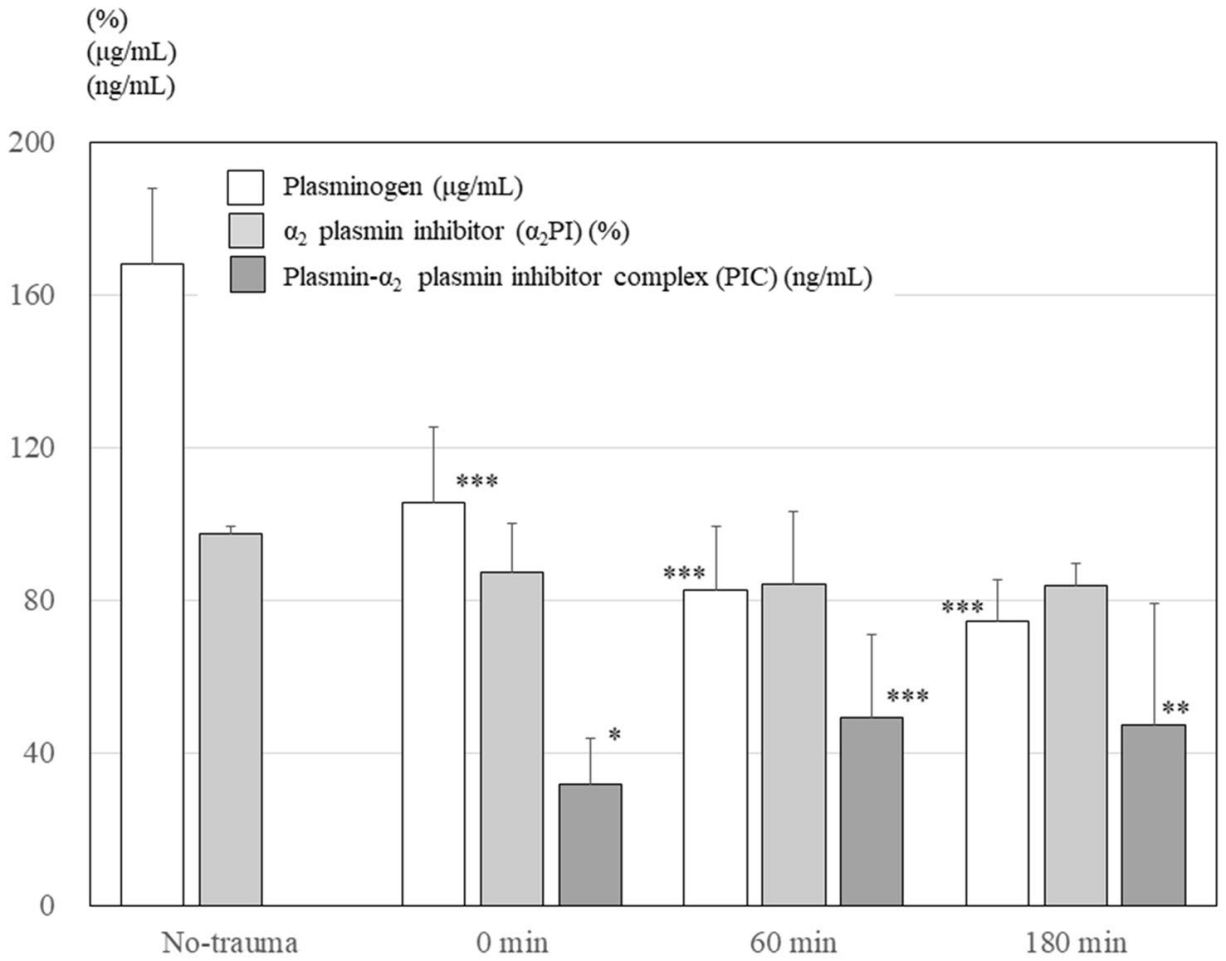
Changes in plasminogen, α_2_ plasmin inhibitor, and plasmin-α_2_ plasmin inhibitor complex levels in the plasma. The plasminogen level decreased immediately after trauma (*P* < 0.001, one-way ANOVA). The α_2_PI levels decreased slightly after trauma. Although PIC was not detected before trauma (the no-trauma group), PIC increased immediately after trauma (*P* <  0.001, one-way ANOVA). However, α2PI levels slightly decreased immediately after trauma but were not significant. α_2_PI, α_2_ plasmin inhibitor; PIC, plasmin-α_2_ plasmin inhibitor complex. ***P <  0.001 using Dunnett’s test for the the no-trauma group group; **P < 0.01 using Dunnett’s test for the no-trauma group; *P < 0.05 using Dunnett’s test for t the no-trauma group.

Figure 5 presents changes in plasminogen, α_2_PI, and PIC levels in the plasma. The plasminogen level significantly decreased immediately following trauma (*P* < 0.001, one-way ANOVA), whereas the PIC level, which is indicative of plasmin production, significantly increased immediately following trauma (*P* < 0.001, one-way ANOVA). However, α_2_PI levels slightly decreased immediately following trauma but did not reach statistical significance.

## Discussion

In the present study, we used a rat model of severe blunt trauma to elucidate the temporal changes in the balance between activation and suppression of the fibrinolytic system in the hours following severe trauma. Coagulation activation, which was indicated by elevated soluble fibrin levels, was observed immediately following trauma and gradually enhanced. Although fibrinolytic activation was observed immediately following the trauma, it did not continue. The fibrinolytic system was intensely suppressed based on an exponential increase in plasma PAI-1 levels.

In the present study, immediately following severe blunt trauma, the fibrinolytic balance tipped significantly toward fibrinolytic activation, compared with the fibrinolytic status before trauma. The fibrinolytic potential in the plasma is governed by the balance between PAI-1 and tPA levels in the plasma, but not by the balance between active and total tPA^11^. Elevation in tPA levels in the plasma immediately following trauma overcame the inactivating effect of PAI-1 and induced fibrinolytic activation. In Fig. 3, we clearly presented this change of balance between fibrinolytic activation and suppression as a ratio of active tPA and active PAI-1 in the plasma. Large amounts of tPA are stored in granules of vascular endothelial cells and are released acutely for a rapid increase in blood active tPA levels by various stimulators, including coagulation activation^10^. The active tPA released into the plasma was inactivated by binding to the active PAI-1 to form tPA·PAI-1 complex, resulting in the increase of the total tPA. From the PAI-1 side, the active PAI-1 was also released into the plasma and bound to active tPA to form tPA·PAI-1 complex, which was inactivated, resulting in total PAI-1 increase. However, active PAI-1 decreased consumptively since the amount of released active tPA was greater than the amount of released active PAI-1. As a result, the active tPA and active PAI-1 ratios significantly increased compared to the ratio before trauma (no trauma group), and the balance between activation and suppression of the fibrinolytic system tipped toward fibrinolytic activation. The fibrinolytic potential on vascular endothelial cell surfaces is also governed by the balance between active PAI-1 and active tPA and reflects the plasma concentrations of active PAI-1 and active tPA^11,28^. However, the vestige of fibrinolytic activation disappeared quickly and was not observed for more than 60 min after trauma.

In the present study, fibrinolytic activation produced plasmin from plasminogen and was also observed immediately after trauma as plasminogen depletion and PIC elevation. The significant elevation of the PIC level immediately following trauma indicated massive production of plasmin. Thus, the significant depletion of plasminogen level immediately following trauma would result from consumption owing to massive plasmin production. Elevated plasma PIC levels were continuously observed until 180 min after trauma. However, although the half-life of free active plasmin is quite short at less than 100 ms, the half-life of PIC is about 4.5 h^12,29,30^. Moreover, no further depletion of plasminogen was observed more than 60 min following trauma. Therefore, the elevated PIC levels more than 60 min after trauma were probably a remnant of the PIC increase immediately following trauma.

In the present study, although total tPA levels in plasma more than 60 min after trauma did not differ from those before trauma, total and active PAI-1 levels increased exponentially after trauma. This difference between tPA and PAI-1 levels in the plasma results from the following two points: 1) The mRNA expression levels of both tPA and PAI-1 gradually increased after trauma; however, the upward trend of the PAI-1 mRNA expression level was significantly stronger than that of tPA and 2) there are large differences between the in vivo half-lives of tPA and PAI-1^31,32^. The half-life of tPA is several minutes, but the half-life of PAI-1 is several hours^31,32^. Therefore, plasma PAI-1 levels, but not tPA levels, gradually increased.

Although tPA has central roles in the fibrinolytic activation to convert the plasminogen into the plasmin, urokinase-type plasminogen activator (uPA) can also activate plasminogen into plasmin^13^. Few studies have reported on the elevation of uPA levels following trauma^33–35^. Although the uPA levels elevated similar to tPA following trauma, certain characteristics were different. (1) Although the baseline concentrations of uPA and tPA were not so different^13^, the active uPA concentration in plasma following severe trauma was one order of magnitude less than that of active tPA^34^, (2) Although the peak of tPA elevation was immediately following trauma, the peak of uPA elevation was several hours following trauma^33^. In the present study, we did not evaluate the temporal changes of uPA, which is one of the limitations. However, as mentioned in previous studies, the plasma concentration of active uPA is much lower than that of active tPA during the first several hours after trauma^34^, and PAI-1 can inhibit both tPA and uPA^13^. The present study showed that the plasma level of active PAI-1 increases intensely after trauma. Therefore, we speculate that uPA will not significantly affect the fibrinolytic status during the first several hours after trauma.

There have been only three studies regarding fibrinolytic regulations by TAFI following trauma^36–38^. Lustenberger et al. indicated that low TAFIa levels on arrival at emergency department were associated with coagulopathy following trauma^37^. However, detail the pathophysiological changes of the TAFIa levels were unclear following trauma^36–38^. In the present study, we did not evaluate the temporal changes of TAFI and TAFIa levels, which is one of the limitations to this study.

Although many clinical studies have reported hyperfibrinolysis after severe trauma, few clinical studies have examined the balance between activation and suppression of fibrinolysis. In previous clinical studies, similar to our results, massive release of tPA overwhelmed the suppression effects of PAI-1 in the plasma immediately following severe trauma^5,39,40^. Coats et al. indicated that PAI-1 levels in the plasma gradually increased and induced an anti-fibrinolytic state several hours after severe trauma, similar to our results^40^. However, the rat models in the present study did not complicate the massive bleeding. Massive bleeding induces hypoperfusion and activates further tPA release^1,9^. Therefore, in patients with trauma with massive bleeding, fibrinolytic activation immediately following trauma will be further accelerated^1,3,37^. Several studies reported the fibrinolytic phenotypes, which included hyperfibrinolysis, and physiologic and fibrinolytic shutdown, on arrival at emergency departments^41–43^. The fibrinolytic phenotypes were strongly related with the transfusion amounts, frequency of organ failure, and mortality rate^41–43^. However, the pathophysiologic differences that induced each fibrinolytic phenotype have been unclear^41–43^.

Recent guidelines on the management of major bleeding in severe trauma suggest that tranexamic acid should be administered to patients as early as possible and continuously infused over 8 h^44,45^. This suggestion is based on large international randomized control trials^6–8^. In the trials, although tranexamic acid administrations improved outcomes in severe trauma patients, there was no pathophysiological evidence for tranexamic acid administration^6–8^. As mentioned in the previous paragraph, the fibrinolytic phenotypes included fibrinolytic shutdown, which were observed on arrival at emergency departments^41–43^. Therefore, selective use of tranexamic acid would be needed based on the fibrinolytic phenotypes^41–43^. Based on the results of the present study, the early administration of tranexamic acid is appropriate and necessary to suppress the fibrinolytic activation immediately following severe trauma. Furthermore, late administration of tranexamic acid can paradoxically increase plasminogen activation by uPA, which elevates at a later time point following trauma, and could increase bleeding^33,46^. Therefore, late time administration of tranexamic acid, including continuous infusion during several hours after trauma may be unnecessary because the fibrinolytic activation is quickly and intensely suppressed by exponential increase of PAI-1.

## Conclusion

Immediately following severe blunt trauma, the massive release of tPA overwhelmed the suppressive effects of PAI-1 in the plasma. However, the production of PAI-1 increased gradually in various organs, and plasma PAI-1 levels increased exponentially. Therefore, immediately following trauma, the fibrinolytic system was activated; however, its activation was quickly and intensely suppressed. Furthermore, the results of this study suggest that although the early administration of tranexamic acid is essential to inhibit fibrinolytic activation immediately following severe trauma, continuous infusion for several hours after trauma is not necessary.

## Supplementary Information

Supplementary Figures.

## References

[1] Hayakawa M (2017). Pathophysiology of trauma-induced coagulopathy: disseminated intravascular coagulation with the fibrinolytic phenotype. J. Intensive Care.

[2] Moore HB, Moore EE (2020). Temporal changes in fibrinolysis following injury. Semin. Thromb. Hemost..

[3] Hayakawa M (2017). Hyperfibrinolysis in severe isolated traumatic brain injury may occur without tissue hypoperfusion: a retrospective observational multicentre study. Crit. Care.

[4] Hayakawa M (2016). High D-dimer levels predict a poor outcome in patients with severe trauma, even with high fibrinogen levels on arrival: a multicenter retrospective study. Shock.

[5] Chapman MP (2016). Overwhelming tPA release, not PAI-1 degradation, is responsible for hyperfibrinolysis in severely injured trauma patients. J. Trauma Acute Care Surg..

[6] Collaborators C-T (2010). Effects of tranexamic acid on death, vascular occlusive events, and blood transfusion in trauma patients with significant haemorrhage (CRASH-2): a randomised, placebo-controlled trial. Lancet.

[7] Collaborators C-T (2011). The importance of early treatment with tranexamic acid in bleeding trauma patients: an exploratory analysis of the CRASH-2 randomised controlled trial. Lancet.

[8] Effects of tranexamic acid on death (2019). disability, vascular occlusive events and other morbidities in patients with acute traumatic brain injury (CRASH-3): a randomised, placebo-controlled trial. Lancet.

[9] Gando S, Hayakawa M (2016). Pathophysiology of trauma-induced coagulopathy and management of critical bleeding requiring massive transfusion. Semin. Thromb. Hemost..

[10] Kruithof EK, Dunoyer-Geindre S (2014). Human tissue-type plasminogen activator. Thromb. Haemost..

[11] Urano T (2019). Recognition of plasminogen activator inhibitor Type 1 as the primary regulator of fibrinolysis. Curr. Drug Targets.

[12] Reed GL, Houng AK, Singh S, Wang D (2017). α2-Antiplasmin: new insights and opportunities for ischemic stroke. Semin. Thromb. Hemost..

[13] Rijken DC, Lijnen HR (2009). New insights into the molecular mechanisms of the fibrinolytic system. J. Thrombos. Haemostasis.

[14] Nachman RL, Hajjar KA, Silverstein RL, Dinarello CA (1986). Interleukin 1 induces endothelial cell synthesis of plasminogen activator inhibitor. J. Exp. Med..

[15] Hou B (2004). Tumor necrosis factor alpha activates the human plasminogen activator inhibitor-1 gene through a distal nuclear factor kappaB site. J. Biol. Chem..

[16] Huebner BR (2018). Thrombin stimulates increased plasminogen activator inhibitor-1 release from liver compared to lung endothelium. J. Surg. Res..

[17] Konkle BA (1992). Plasminogen activator inhibitor-1 messenger RNA expression is induced in rat hepatocytes in vivo by dexamethasone. Blood.

[18] Kawai S, Takagi Y, Kaneko S, Kurosawa T (2011). Effect of three types of mixed anesthetic agents alternate to ketamine in mice. Exp. Anim..

[19] Noble RL, Collip JB (1942). A quantitative method for the production of experimental traumatic shock without hæmorrhage in unanæsthetized animals. Q. J. Exp. Physiol. Cogn. Med. Sci..

[20] Hayakawa M (2015). Noble-collip drum trauma induces disseminated intravascular coagulation but not acute coagulopathy of trauma-shock. Shock.

[21] Hayakwa M (2020). Microparticles and nucleosomes are released from parenchymal cells destroyed after injury in a rat model of blunt trauma. Clin. Appl. Thrombosis Hemostasis.

[22] Yan Z (2017). Activation of caspase-12 at early stage contributes to cardiomyocyte apoptosis in trauma-induced secondary cardiac injury. Acta Physiol. Sinica.

[23] Ma S (2017). Mitigation effect of proanthocyanidin on secondary heart injury in rats caused by mechanical trauma. Sci. Rep..

[24] Li X (2017). Curcumin ameliorates cardiac dysfunction induced by mechanical trauma. Eur. J. Pharmacol..

[25] Feng Y (2013). Insulin alleviates posttrauma cardiac dysfunction by inhibiting tumor necrosis factor-α-mediated reactive oxygen species production. Crit. Care Med..

[26] Campbell B, Chuhran C, Lefer AM (2000). Vascular endothelial growth factor attenuates trauma-induced injury in rats. Br. J. Pharmacol..

[27] Winer J, Jung CKS, Shackel I, Williams PM (1999). Development and validation of real-time quantitative reverse transcriptase-polymerase chain reaction for monitoring gene expression in cardiac myocytesin vitro. Anal. Biochem..

[28] Suzuki Y, Mogami H, Ihara H, Urano T (2009). Unique secretory dynamics of tissue plasminogen activator and its modulation by plasminogen activator inhibitor-1 in vascular endothelial cells. Blood.

[29] Booth NA, Bennett B (1982). Plasmin–alpha 2-antiplasmin complex as an indicator of in vivo fibrinolysis. Br. J. Haematol..

[30] Chandler WL, Alessi MC, Aillaud MF, Vague P, Juhan-Vague I (2000). Formation, inhibition and clearance of plasmin in vivo. Haemostasis.

[31] Jankun J (2012). Remarkable extension of PAI-1 half-life surprisingly brings no changes to its structure. Int. J. Mol. Med..

[32] Chandler WL (1997). Clearance of tissue plasminogen activator (TPA) and TPA/plasminogen activator inhibitor type 1 (PAI-1) complex: relationship to elevated TPA antigen in patients with high PAI-1 activity levels. Circulation.

[33] Hijazi N (2015). Endogenous plasminogen activators mediate progressive intracerebral hemorrhage after traumatic brain injury in mice. Blood.

[34] Cardenas JC (2014). Elevated tissue plasminogen activator and reduced plasminogen activator inhibitor promote hyperfibrinolysis in trauma patients. Shock.

[35] Griemert EV (2019). Plasminogen activator inhibitor-1 augments damage by impairing fibrinolysis after traumatic brain injury. Ann. Neurol..

[36] Relja B (2013). Thrombin-activatable fibrinolysis inhibitor (TAFI) is enhanced in major trauma patients without infectious complications. Immunobiology.

[37] Lustenberger T (2013). Activated thrombin-activatable fibrinolysis inhibitor (TAFIa) levels are decreased in patients with trauma-induced coagulopathy. Thromb. Res..

[38] Hayakawa M (2012). A low TAFI activity and insufficient activation of fibrinolysis by both plasmin and neutrophil elastase promote organ dysfunction in disseminated intravascular coagulation associated with sepsis. Thromb. Res..

[39] Moore HB (2017). Fibrinolysis shutdown is associated with a fivefold increase in mortality in trauma patients lacking hypersensitivity to tissue plasminogen activator. J. Trauma Acute Care Surg..

[40] Coats TJ, Morsy M (2020). Biological mechanisms and individual variation in fibrinolysis after major trauma. Emerg Med. J..

[41] Moore HB (2014). Hyperfibrinolysis, physiologic fibrinolysis, and fibrinolysis shutdown: the spectrum of postinjury fibrinolysis and relevance to antifibrinolytic therapy. J. Trauma Acute Care Surg..

[42] Moore HB (2019). Does tranexamic acid improve clot strength in severely injured patients who have elevated fibrin degradation products and low fibrinolytic activity, measured by thrombelastography?. J. Am. Coll. Surg..

[43] Stettler GR (2019). Redefining postinjury fibrinolysis phenotypes using two viscoelastic assays. J. Trauma Acute Care Surg..

[44] Rossaint, R. *et al.* The European guideline on management of major bleeding and coagulopathy following trauma: fourth edition. *Critical care* **20**, 100, doi:10.1186/s13054-016-1265-x (2016). 10.1186/s13054-016-1265-x PMC4828865 27072503

[45] Cannon JW (2017). Damage control resuscitation in patients with severe traumatic hemorrhage: a practice management guideline from the Eastern Association for the Surgery of Trauma. J Trauma Acute Care Surg.

[46] Medcalf RL (2015). The traumatic side of fibrinolysis. Blood.

