# Supplementary figures and images for: Fibrinolytic system activation immediately following trauma was quickly and intensely suppressed in a rat model of severe blunt trauma
Source: Sci Rep. 2021 Oct 13;11:20283. doi: 10.1038/s41598-021-99426-2 (PMC8514435; doi:10.1038/s41598-021-99426-2)

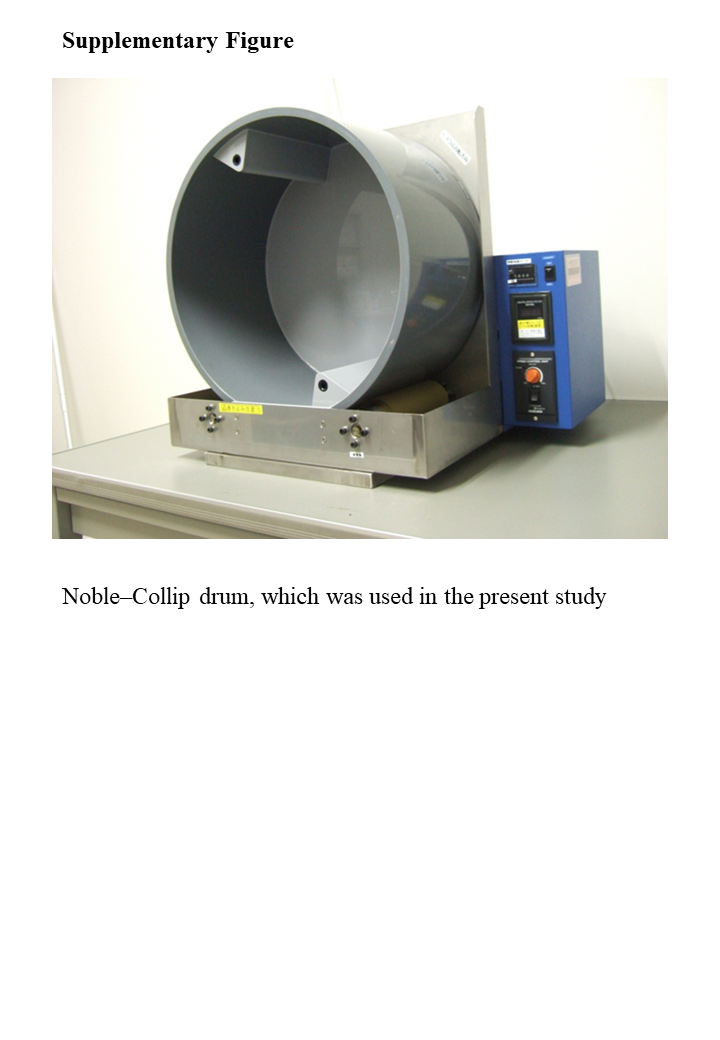

Supplement: Supplementary file 1 — Supplementary Figures. [file 41598_2021_99426_MOESM1_ESM.tif]
